# Abnormalities in gamma-band responses to language stimuli in first-degree relatives of children with autism spectrum disorder: an MEG study

**DOI:** 10.1186/1471-244X-12-213

**Published:** 2012-11-29

**Authors:** Kristina L McFadden, Susan Hepburn, Erin Winterrowd, Gwenda L Schmidt, Donald C Rojas

**Affiliations:** 1 Department of Psychiatry at the University of Colorado Denver Anschutz Medical Campus, 13001 E. 17th Place, Aurora, CO, 80045, USA; 2 JFK Partners at the University of Colorado Denver Anschutz Medical Campus, 13121 E. 17th Ave, Aurora, CO, 80045, USA; 3 Department of Psychology, University of Wisconsin Oshkosh, 800 Algoma Blvd, Oshkosh, WI, 54901, USA; 4 Department of Psychology, Hope College, 35 E 12th St, Holland, MI, 49423, USA

## Abstract

**Background:**

Synchronous neural oscillatory activity in the gamma range (30–80 Hz) has been shown to be abnormal in individuals with autism spectrum disorders (ASD) and their first-degree relatives in response to simple auditory stimuli. Gamma-band abnormalities in ASD probands have been seen in response to language stimuli, but this has not been investigated in first-degree relatives. This is of particular interest given that language impairments are a core symptom of ASD and may be part of the broad autism phenotype (BAP) seen in relatives.

**Methods:**

Magnetoencephalography recordings during a continuous word recognition task were obtained for 23 parents of a child with ASD (pASD) and 28 adult control participants. Total and evoked gamma-band activity, as well as inter-trial phase-locking factor (PLF), were measured in response to the task. Beta-band activity was also measured, due to its suggested role in language processing. Participants completed a series of language measures to assess the relationship between brain activity and language function, and lateralization of task-related activity was assessed.

**Results:**

The pASD group showed increased evoked gamma and beta activity, while controls had decreased evoked activity. Additionally, while both groups showed a reduction in total gamma power (commonly seen in language tasks), this reduction was more prominent in the control group. The pASD group demonstrated significantly worse performance on a measure of phonology compared to controls. Significant but distinct relationships were found between gamma/beta activity and language measures within the two groups. In addition, while the overall task generally elicited left lateralized responses, pASD showed greater left lateralization than controls in some regions of interest.

**Conclusions:**

Abnormalities in oscillatory responses to language were seen in pASD that are consistent with previous findings in ASD probands. Gamma-band responses to language stimuli have not previously been assessed in first-degree relatives of ASD probands and these findings are supportive of gamma-band activity as a heritable, neurophysiological biomarker of ASD. The possible relationship seen between language function and neural activity in the current study should be investigated further to assess if oscillatory response abnormalities may contribute to behavioural manifestations of the BAP.

## Background

Autism spectrum disorders (ASD) are characterized by difficulties in social interaction, communication and restricted interests [1] and are relatively common in the United States, with prevalence rates estimated to be as high as 1 in 110 children [2]. As diagnosis of ASD currently relies on behavioral observations and caregiver interviews alone, the discovery of physiological markers of ASD would provide objective markers of the condition, which would facilitate further research and treatment.

Synchronous neural activity in the gamma range (30–80 Hz) has been implicated as a neurophysiological biomarker in ASD and can be reliably recorded via magnetoencephalography (MEG). Gamma-band activity is either phase-locked to the stimulus (evoked responses) or not phase-locked (induced responses); together, evoked and induced power make up total gamma-band power [3], although many researchers consider the phase-locked and non-phase-locked components together, terming it induced power [4]. In this conception, induced power and total power are equivalent terms. While the precise role of gamma-band activity is unclear, it has been implicated in a wide range of processes such as attention [5-7], working memory [8,9], early processing of sensory information [10,11], language [12-15], and perceptual binding [16,17]. This suggests that gamma-band oscillations are produced across many areas of the cerebral cortex and are sensitive to a wide variety of task manipulations. This is particularly relevant to ASD because many of these processes are thought to be abnormal in this condition [18-24].

Grice et al. [25] first reported gamma-band abnormalities in ASD. They found induced gamma activity to be greater to visual presentation of upright faces compared to inverted faces in adult control participants, but did not see the gamma-band inversion effect in a group of adults with ASD. Previous MEG studies have also found gamma-band abnormalities in response to auditory tone or click-train stimuli in children and adults with ASD [26-28], further implicating it as a biomarker of ASD. Specifically, children with ASD show reduced evoked gamma-band activity [26] and reduced gamma-band phase-locking to stimuli [28] compared to controls. Adults with ASD also show this reduction in evoked activity and phase-locking [27]. Braeutigam et al. [29] reported gamma-band activity abnormalities in adults with ASD during a more complex auditory task involving listening to sentences ending in logical or illogical words. While both groups showed increased evoked gamma activity, the ASD group demonstrated a more complex pattern of increased activation that was stronger and more widespread across the time window.

The gamma-band abnormalities (i.e., reduced evoked gamma activity) seen in ASD in response to simple and steady-state auditory stimuli have also been found in parents of children with ASD (pASD), implicating gamma-band activity as a potentially heritable ASD biomarker [27,30]. ASD is thought to be highly heritable, although there is inconsistency in heritability estimates. While some have estimated heritability to be as high as 90% [31,32], recent work has suggested it could be closer to 38% [33]. Nonetheless, subclinical features of ASD are often seen in first-degree relatives of those with ASD, a phenomenon referred to as the broad autism phenotype (BAP) [34,35]. Identification of physiological markers of ASD seen as part of the BAP could help in determining underlying causes of ASD symptoms, as well as advancing ASD research and treatment monitoring.

The current study extends previous research by assessing gamma-band abnormalities in pASD in response to auditory language stimuli, which has not previously been investigated. While Braeutigam et al. [29] found increased evoked gamma-band responses to spoken sentences in adults with ASD, this has not been studied in first-degree relatives. In previous studies with simpler auditory stimuli (e.g., pure tone and modulated noise stimuli), both individuals with ASD [26,27] and pASD [27,30] demonstrated decreases in evoked gamma power compared to controls. Previous studies of gamma-band activity during language stimulation commonly demonstrate event-related desynchronization (ERD) in response to language, suggested to reflect semantic processing [13,36-38]. As there are differences between findings using different kinds of stimulation and experimental paradigms in ASD, it is important to know if findings in pASD are also contextually specific. The current study investigates whether the gamma-band abnormalities seen in pASD in response to language stimuli are similar to those seen in ASD, with the goal of further understanding and refining the biomarker. To assess this, participants completed an auditory continuous word recognition (CWR) task [39] during MEG recording. By incorporating language into the task, the gamma-band abnormalities in pASD can be evaluated more thoroughly than has been done previously. In addition, this task is of particular interest in this population because language and communication difficulties are a core deficit in ASD [1], and can be seen as part of the BAP [35,40,41]. Therefore, the current study also explored whether gamma-band abnormalities to language stimuli are related to behavioral measures of language.

While much of the research on neural oscillations in ASD has focused on gamma-band activity, synchronous activity in the beta band (13–30 Hz) is also relevant to the current study. Neural oscillations in the beta band have been implicated in top-down cognitive control [42,43] and may be involved in language processing [12,37,44,45]. Few studies have investigated beta oscillations in ASD, but increases in beta power have been found in children with ASD compared to typically developing controls [46]. The current study will also investigate group differences in beta-band activity.

In summary, findings from the current study will further elucidate gamma-band abnormalities seen in pASD. If these abnormalities are related to simple sensory aspects of the presented stimuli, results should be consistent with those of previous studies using tone and click-train stimuli, with pASD demonstrating reduced evoked gamma-band activity compared to controls. However, if gamma-band abnormalities are reflective of higher order cognitive processes, the pattern of group differences will likely be similar to those seen in Braeutigam et al. [29], with pASD showing a more complex pattern of *increased* evoked gamma activity compared to controls. Group differences in beta-band activity will also be assessed, as beta-band activity may play a role in language processing. Additionally, the current study will investigate the relationship between behavioral measures of language and gamma-band activity, assessing a possible link between abnormal gamma-band activity and behavioral manifestations of the BAP. This will also be explored for beta-band activity. Lastly, the study will examine lateralization of brain activity during the task, as abnormal lateralization of language function has been found in ASD [29,47] and the CWR task has previously been used to assess language lateralization [39]. As previous studies have suggested that individuals with ASD tend to show right language lateralization rather than the left lateralization usually seen in typically developing individuals [29,47], we hypothesized that pASD would show less left lateralization than controls.

## Methods

### Participant characteristics

Twenty-three parents (8 male, 15 female; mean age = 35.84 +/− 9.99 years) of a child with an Autism Spectrum Disorder (ASD) participated in the study. Each parent had one child meeting DSM-IV criteria for ASD, as determined by consensus of the Autism Diagnostic Observation Schedule (ADOS [48]), the Autism Diagnostic Interview, Revised (ADI-R [49]) and DSM-IV diagnosis by a clinical psychologist. Only one parent per family participated in the study. Twenty-eight healthy adults (12 male, 16 female; mean age = 38.70 +/− 6.29 years) with no personal or family history of developmental disorder participated as a control group. Participants were recruited via clinical referral (for parents) or fliers/mass email postings (for controls).

### Procedure

Participants provided informed consent and all procedures were in accordance with the guidelines of the Colorado Multiple Institutional Review Board. Following consent, demographic measures were collected, including age, gender, ethnicity, socioeconomic status (as determined by the Hollingshead Four-Factor Index of Social Status (SES), family weighted total score [50]), and handedness, assessed with the Annett handedness questionnaire [51]. To obtain an overall measure of cognitive ability, the Wechsler Abbreviated Scale of Intelligence (WASI; Psychological Corporation, 1999) was used to evaluate IQ in both groups.

#### Language measures

To assess the relationship between language performance and neural activity, participants completed a battery of language measures prior to MEG recording. The Peabody Picture Vocabulary Test (PPVT-III [52]) was administered to capture receptive language, with the Expressive Vocabulary Test (EVT [53]) and the Verbal Fluency subtest from the Delis Kaplan Executive Function System (DK-EFS [54]) used as measures of expressive language. Additionally, the Figurative Language subtest from the Test of Language Competence-Expanded Edition (TOLC-E [55]) assessed figurative language, and the Nonword Repetition subtest of the Comprehensive Test of Phonological Processing (CTOPP [56]) captured phonological processing.

Independent-samples t-tests were used to assess group differences (pASD vs. control) in demographic, IQ, and language measures, using SPSS 17.0 (SPSS, Inc., Chicago, IL). Scores for IQ and language measures were standardized according to manufacturer recommendations and the alpha criterion was set at .05 (two-tailed).

#### Continuous word recognition (CWR) task

To assess neural activity in response to language stimuli, participants completed the CWR task during MEG recording. Prior to MEG set-up, participants were given a visual list of target words and instructed to familiarize themselves with the words so they could recognize them during the task. During MEG recording, participants listened to the spoken words presented via Eprime version 1.3 (Psychology Software Tools, Inc., Pittsburgh, PA) through headphones at 80 dB SPL (with no visual representation of the words). Prior to recording, all participants demonstrated hearing abilities within normal limits (< 20 dB HL) using the method of constant stimuli. CWR task stimuli consisted of abstract English nouns, with 33 target words and 30 distractor words [39]. A recording of a native English speaker (male voice) using a flat intonation was used in stimuli presentation. The words had a mean duration of 450 ms (range 300–750 ms), and were digitized using a sampling rate of 22,500 Hz, with 16-bit resolution. Participants were instructed to slightly lift their dominant index finger after hearing the target words, but not the distractor words. Each target word was presented three times during the task (total of 99 target words) and each distractor word was presented once (total of 30 distractor words). The onset of stimulation was noted with triggers in the data corresponding to the beginning of each word. The interstimulus interval between word presentation randomly varied between 3000 and 4000 ms.

#### MEG data acquisition

MEG data were acquired with a 4D Neuroimaging (San Diego, CA) Magnes WH3600 neuromagnetometer system with 248 axial first-order gradiometers. Recordings were made with participants supine in a custom-built magnetically-shielded room. Prior to MEG recording, the location and orientation of the MEG coils relative to each subject’s head were determined by digitizing a set of fiducial reference points on the head using a magnetic digitizer (Polhemus 3SPACE). Left and right preauricular points and the nasion (as defined by the International 10–20 electrode system [57]) were digitized as reference points and the shape of each participant’s head was digitized for use in constructing a volume conductor model for source localizations. Stimuli were delivered via foam insert earphones (E.A.R., Cabot Safety Co., Indianapolis, IN) and data were collected at a sampling rate of 678.17 Hz, with an epoch window of 950 ms (150 ms pre-stimulus and 800 ms post-stimulus).

### Data processing and analyses

#### MEG data: pre-processing and source analysis

Following data acquisition, all epochs with values exceeding +/− 2500 fT were rejected from further analysis to exclude trials with eyeblinks and movement artifacts. In data with additional eyeblink artifacts, independent components analysis (ICA; EEGLAB [58]) was used to separate and remove any remaining eyeblink signal, while minimizing loss of usable trials. Analyses focused on responses to target words; trials for the distractor words were not included.

Source analysis was performed in Statistical Parametric Mapping SPM8 (Wellcome Trust Centre for Neuroimaging, London, UK) implemented in MATLAB (2009b; MathWorks, Inc., Natick, MA). Following coregistration of MEG fiducials with the SPM8 standard MRI template and the construction of a forward model (single sphere [59]), source localization used a Bayesian cortically constrained group minimum norm inversion (with multiple sparse priors (MSP) used for priors) [60,61]. All subjects’ data were entered into the inverse solution simultaneously in the SPM8 group inversion process, which results in a common source space across subjects. Using the image files created from the group inversion, a one-sample t-test across the two groups was conducted in SPM8 to determine regions of interest (ROIs) in which activity during the task survived multiple comparison correction (see [61] for details), using a false discovery rate (FDR) of q = .05 [62]. Because the later language-related response to the task was of primary interest, the inversion and subsequent t-test focused on the 200–800 ms post-stimulus time window. Montreal Neurological Institute (MNI) coordinates for each of the areas surviving multiple comparison correction in left and right hemispheres were determined (see Table 1). As anticipated, areas commonly associated with auditory and/or language tasks [63-65] were found to be active, in both left and right hemispheres: auditory cortex, supramarginal gyrus (SMG), fusiform gyrus (FFG), lateral occipital cortex (LOC), and dorsolateral prefrontal cortex (DLPFC).

**Table 1:** MNI coordinates for each region of interest used in source space projection

**Label**	**MNI coordinates**			**t-value**
DLPFC				
Left hemisphere	−24	34	28	9.89
Right hemisphere	20	30	34	10.46
Auditory cortex				
Left hemisphere	−58	0	2	14.42
Right hemisphere	60	–2	0	12.80
SMG				
Left hemisphere	−56	–30	30	11.35
Right hemisphere	60	–28	38	10.95
FFG				
Left hemisphere	−36	–64	–14	26.60
Right hemisphere	36	–62	–18	26.37
LOC				
Left hemisphere	−46	–66	–2	16.74
Right hemisphere	42	–70	–10	18.12

*MNI* Montreal Neurological Institute, *DLPFC* dorsolateral prefrontal cortex, *SMG* supramarginal gyrus, *FFG* fusiform gyrus, *LOC* lateral occipital cortex

Following multiple comparison correction (FDR), all *p* < .001.

#### MEG data: source space projection and time-frequency analysis

The MNI coordinates for each ROI were used to seed an equivalent current dipole in source space, whose orientation was the normal of the cortical surface at that point. Source space projection (SSP) was done from these points to project the original raw MEG data into source space, creating a series of “virtual sensors”, or dipole waveforms. SSP projects activity to particular locations in the brain based on weights calculated from the pseudo-inverse of the leadfield vector (for details, see [66,67]). Using the MNI coordinates in Table 1, SSP was used to create waveforms for each ROI, using custom MATLAB routines and SPM8 code. Projections were performed on the individual MEG trials rather than the averages for the purpose of calculating time-frequency metrics.

To calculate gamma- and beta-band power for each ROI, data were then transformed to the time-frequency domain using a Morlet wavelet (wave number 6) decomposition [68]. Time-frequency transformation was performed using custom MATLAB routines. Mean total (a.k.a. induced power) and evoked gamma-band power (both expressed relative to baseline as a percentage) and mean phase-locking factor were calculated for each brain hemisphere between 30–50 Hz in two time windows: (1) 30–150 ms post-stimulus, where the transient gamma-band response (tGBR) is expected [3,5,69], and (2) 200–800 ms post-stimulus, to assess a later gamma-band response seen in exploratory analyses. Beta-band (13–30 Hz) total and evoked power were determined for the same time windows.

Statistical analyses were performed in SPSS. A mixed design analysis of variance (ANOVA) was used for each ROI, with the following factors: 2 groups (pASD and controls) and 2 brain hemispheres (left and right). Given that there were 5 ROIs used (auditory cortex, SMG, FFG, LOC, and DLPFC), statistical significance was set at *p* = .01, as per Bonferroni correction for an alpha of .05. While the results presented here use SSP, sensor-level analyses were also performed to ascertain that both methods would find similar results. This was the case, although sensor-level results did not survive multiple comparison correction, likely due to SSP conferring a greater signal to noise ratio (see Additional file 1).

#### Lateralization index

The CWR task used in the current study has been used in prior studies to determine lateralization of language function [39]. This is of particular interest in the current population because previous studies have found individuals with ASD to show right language lateralization, whereas typically developing individuals usually demonstrate left language lateralization [29,47]. Lateralization was assessed using the LI-toolbox version 1.1.1 [70] in SPM8, for two purposes: 1) to determine if results replicate prior studies using the CWR task, and 2) to investigate if lateralization abnormalities seen in those with ASD [29,47] are also seen in pASD. The adaptive threshold setting in the LI-toolbox was used and laterality of both the 0–200 ms window (early auditory response) and the 200–800 ms window in each of the five ROIs was analyzed. This was assessed for both total and evoked gamma (30–50 Hz) and beta (13–30 Hz) activity. The LI toolbox designates output of +1 as purely left activation and −1 as purely right activation. Indices between -.01 and 0 and between 0 and .01 were considered to indicate bilateral activation [39]. Following calculation of the laterality index, group differences in lateralization were analyzed in SPSS via independent samples t-tests. One-sample t-tests were conducted across groups to evaluate lateralization overall for each ROI.

#### Regression analyses

For explorative analyses of the relationship between measures of language and neural activity in the ROIs determined above (see Table 1), regression analyses were performed for both gamma- and beta-power. Multivariate regression analyses were performed in SPSS, with language measures of interest regressed on group, neural activity in each ROI, and the interaction between the two. To correct for comparisons in the 5 ROIs, statistical significance was set at .01 for Bonferroni correction of an alpha of .05.

## Results

### Participant characteristics

The pASD and control groups did not significantly differ in age, ethnicity, SES, or handedness (see Table 2).

**Table 2:** Participant characteristics

	**Group**	
**Measure**	**pASD**	**control**
Age (mean years ± SD)	38.70 ± 6.29	35.84 ± 10.00
Gender (% male)	34.8%	42.9%
Ethnicity		
% Caucasian	91.3%	96.4%
% African American	8.7%	3.6%
Handedness score^a^	.79 ± .36	.68 ± .49
SES (Hollingshead)^b^	50.63 ± 9.49	50.07 ± 8.51
Child SCQ score (pASD only)	21.96 ± 4.09	

pASD = parent of a child with ASD; SCQ = Social Communication Questionnaire.

^a^Handedness score from the Annett handedness questionnaire, in which +1 is completely right handed and −1 is completely left-handed.

^b^SES = Socioeconomic status, as assessed with the Hollingshead Four-Factor Index of Social Status.

### IQ/Language measures

There was a significant group difference on the Nonword Repetition subtest of the Comprehensive Test of Phonological Processing (CTOPP), such that the pASD group demonstrated significantly lower scores than did the control group, *t* (48) = 2.94, *p* = .005. There was also a trend toward the pASD group scoring poorer on the Peabody Picture Vocabulary Test (PPVT-III) than the control group, *t* (48) = 1.99, *p* = .052. There were no significant group differences in IQ or any of the other language measures (see Table 3). There is significant overlap between the current sample of pASD and that reported in a previous study of language measures [71] that reported PPVT, CTOPP and IQ measures, so these findings should not be considered completely independent.

**Table 3:** IQ and language measures

**Measure**	**Group**	
	**pASD**	**control**
IQ		
FSIQ	115.00 ± 7.91	117.82 ± 10.31
VIQ	113.57 ± 9.48	113.64 ± 10.53
PIQ	113.30 ± 11.48	117.89 ± 10.59
Nonword Repetition**	6.91 ± 1.82	8.50 ± 1.95
EVT	110.36 ± 11.17	108.79 ± 14.38
Verbal Fluency		
LF	11.64 ± 2.48	10.64 ± 3.16
CF	12.55 ± 2.74	12.04 ± 2.74
CS	12.55 ± 2.42	12.75 ± 2.94
Figurative Language	12.36 ± 1.62	12.18 ± 1.87
PPVT-III*	106.86 ± 9.38	112.39± 10.05

** = *p* < .01; * = *p* < .06 for group comparisons.

*FSIQ* full-scale IQ, *VIQ* verbal IQ, *PIQ* performance IQ, *EVT* Expressive Vocabulary Test, *LF* Letter Fluency, *CF* Category Fluency, *CS* Category Switching (accuracy), *PPVT-III* Peabody Picture Vocabulary Test.

### Gamma-band activity

**Figure 1:**
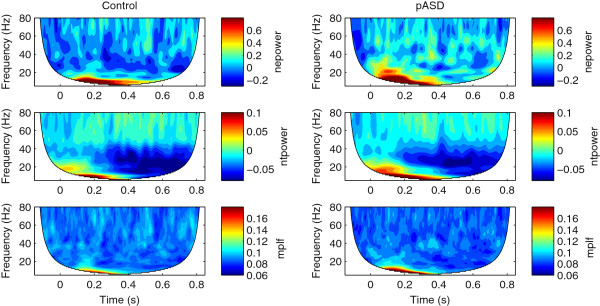
**Left hemisphere time-frequency results.** Time-frequency plots of the grand average across all ROIs for the control group (left column) and the pASD group (right column) for the left hemisphere. The rows show normalized evoked power (nepower), normalized total power (ntpower) and mean phase locking factor (mplf), respectively. The region in white is not analyzed because of incomplete filling of the wavelet near the edges.

**Figure 2:**
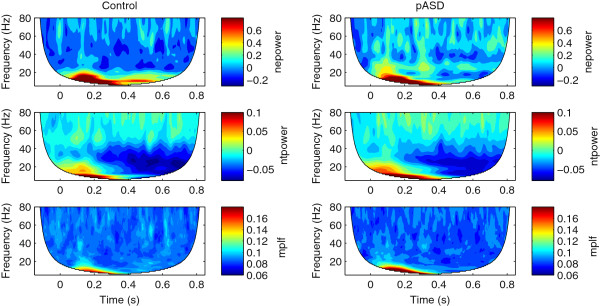
**Right hemisphere time-frequency results.** Time-frequency plots of the grand average across all ROIs for the control group (left column) and the pASD group (right column) for the right hemisphere. The rows show normalized evoked power (nepower), normalized total power (ntpower) and mean phase locking factor (mplf), respectively. The region in white is not analyzed because of incomplete filling of the wavelet near the edges.

Figures 1 and 2 show time-frequency results averaged across all ROIs for both control and pASD groups in left and right hemispheres, respectively. All reported evoked and total gamma-band activity results reflect evoked and total power normalized to baseline.

#### Early tGBR

**Figure 3:**
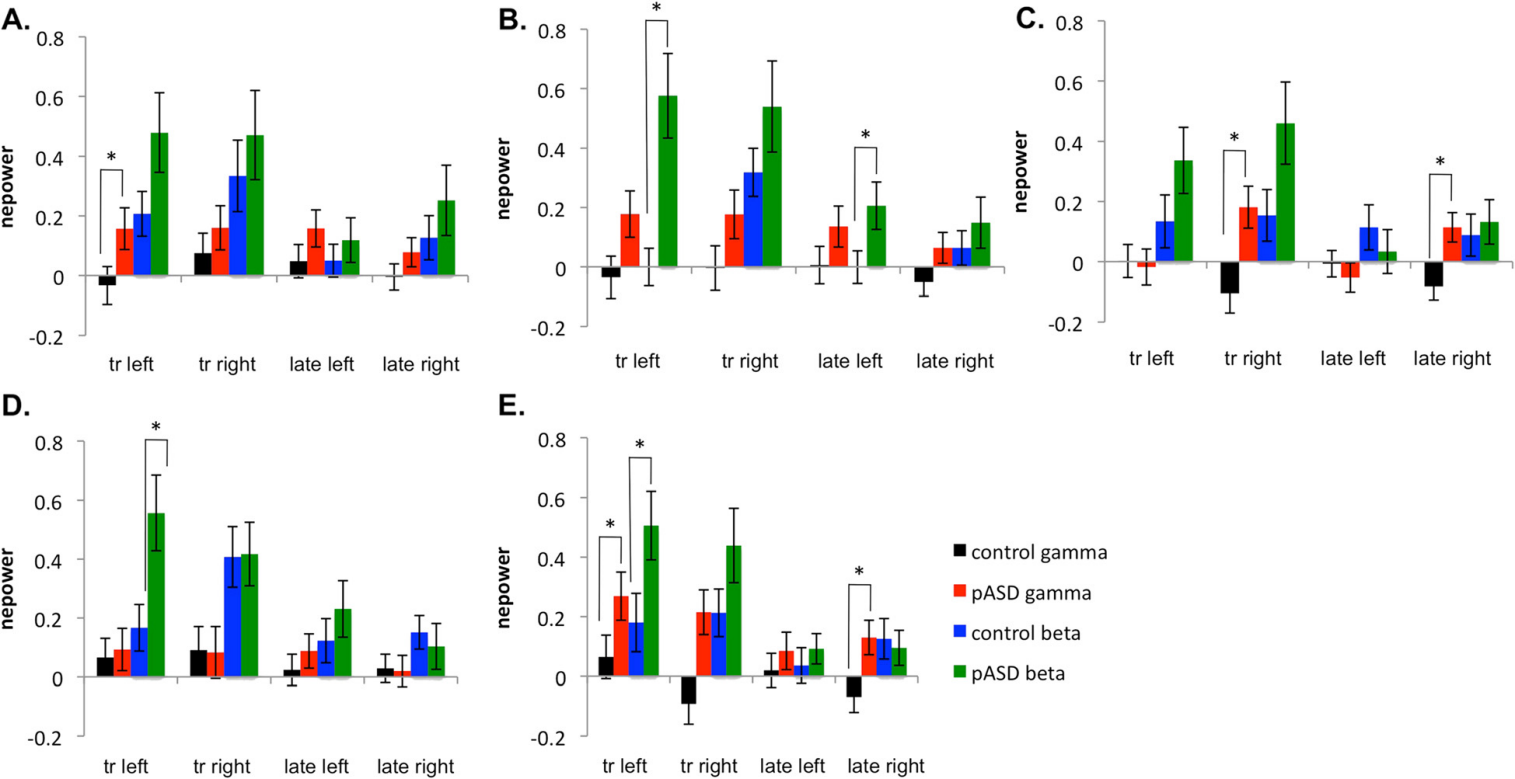
**Evoked power results.** Mean normalized evoked gamma and beta power (nepower) for **A**) auditory cortex, **B**) supramarginal gyrus, **C**) fusiform gyrus, **D**) dorsolateral prefrontal cortex, and **E**) lateral occipital cortex for left and right hemispheres. tr: transient response (30–150 ms post-stimulus), late: late response (200–800 ms post-stimulus).

There was a significant main effect of group in the early transient gamma-band response (tGBR; 30–150 ms post-stimulus) such that pASD showed increased evoked gamma-band power and the control group showed decreased evoked power in lateral occipital cortex (LOC) (*F* (1, 49) = 7.96, *p* = .007) and marginally in supramarginal gyrus (SMG) (*F* (1, 49) = 5.95, *p* = .018),(see Figure 3). There was also a significant interaction between group and hemisphere in evoked power in FFG, *F* (1, 49) = 9.34, *p* = .004. Post-hoc tests found that while there was no significant difference between evoked power in left and right hemispheres in the control group (*p* > .05), there was a significant difference between left and right hemispheres in the pASD group, *p* = .01. While pASD showed a reduction in evoked power in the left hemisphere, an increase was seen in the right hemisphere.

**Figure 4:**
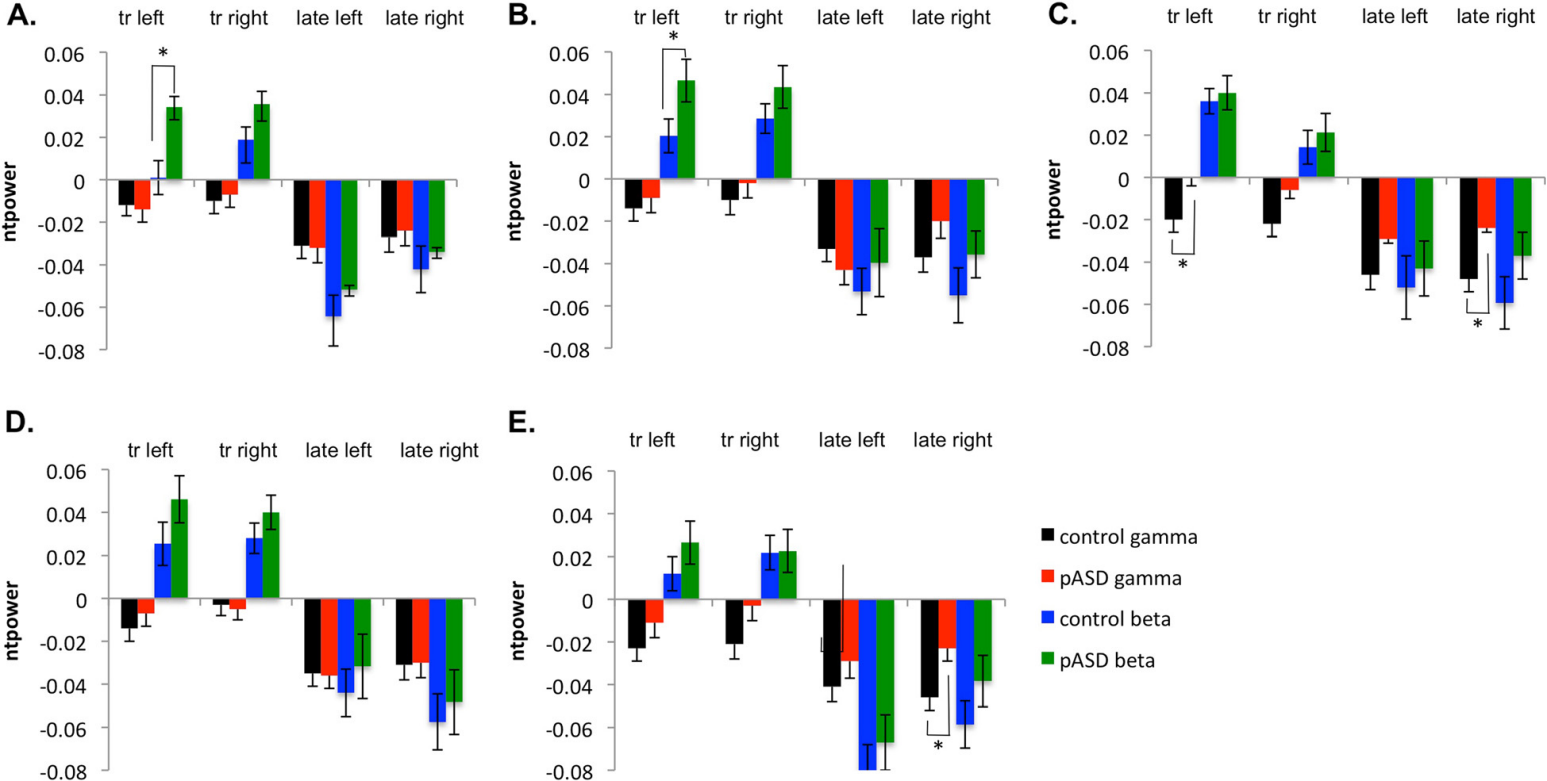
**Total power results.** Mean normalized total gamma and beta power (ntpower) for **A**) auditory cortex, **B**) supramarginal gyrus, **C**) fusiform gyrus, **D**) dorsolateral prefrontal cortex, and **E**) lateral occipital cortex for left and right hemispheres. tr: transient response (30–150 ms post-stimulus), late: late response (200–800 ms post-stimulus).

In early total gamma-band power, there was a significant effect of group in FFG, *F* (1, 49) = 6.97, *p* = .010. Although both groups showed a decrease in total power as compared to baseline, the control group showed a greater decrease than the pASD group (see Figure 4). A similar trend was seen in LOC, with controls showing a greater decrease in total power than pASD, *F* (1, 49) = 3.79, *p* = .057.

#### Late gamma-band activity

A similar pattern was seen in later gamma-band activity (200–800 ms post-stimulus), with pASD demonstrating marginally significant increases in evoked gamma-band power and controls showing decreased power in LOC (*F* (1, 49) = 4.32, *p* = .043) and SMG (*F* (1, 49) = 4.26, *p* = .044) (see Figure 3). There was also a significant group by hemisphere interaction in evoked power in FFG, *F* (1, 49) = 12.18, *p* = .001; there were no significant differences between reduction in evoked power for left and right hemispheres in the control group (*p* > .05), but there was a significant difference between left and right hemispheres in the pASD group, *p* = .006 (i.e., reduced evoked power in left hemisphere accompanied by increased power in right hemisphere).

**Figure 5:**
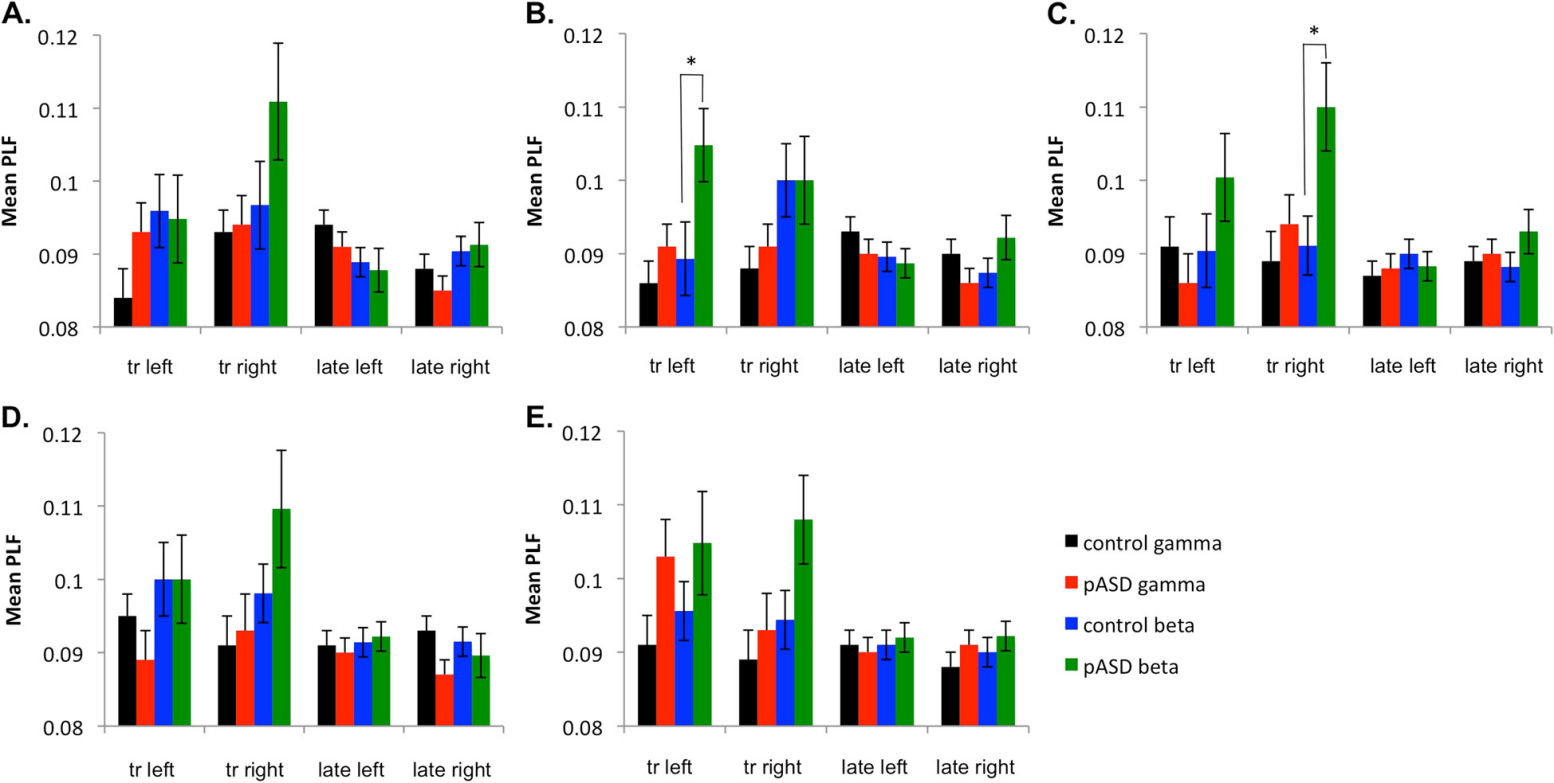
**Phase-locking factor results.** Mean phase locking factor (PLF) for **A**) auditory cortex, **B**) supramarginal gyrus, **C**) fusiform gyrus, **D**) dorsolateral prefrontal cortex, and **E**) lateral occipital cortex for left and right hemispheres. tr: transient response (30–150 ms post-stimulus), late: late response (200–800 ms post-stimulus).

A marginally significant main effect of group was found in total gamma power in FFG, with both groups reduced from baseline, but with the control group showing greater reduction than pASD, *F* (1, 49) = 6.35, *p* = .015 (see Figure 4). The same pattern of greater reduction in controls than pASD was seen in LOC, *F* (1, 49) = 4.18, *p* = .046. There was also a marginally significant group by hemisphere interaction in total power in SMG, *F* (1, 49) = 5.43, *p* = .024. Post-hoc tests found that while there was no significant difference between total power in left and right hemispheres for the control group (*p* > .05), total power was decreased significantly more in the left hemisphere in pASD as compared to the right, *p* = .008.While no significant group differences in phase-locking factor (PLF) were found, a main effect of hemisphere was seen in both auditory cortex (*F* (1, 48) = 12.97, *p* = .001) and marginally for SMG (*F* (1, 48) = 5.37, *p* = .025). This was such that PLF was greater in the left hemisphere than in the right hemisphere across groups (see Figure 5).

### Beta-band activity

Time-frequency results for beta-band activity can be seen in Figures 1 and 2 between 13–30 Hz. All reported evoked and total beta-band activity results reflect evoked and total power normalized to baseline.

#### Early beta-band response

A significant main effect of group was found in evoked beta-band power in SMG; both groups showed increased evoked beta activity, but beta increased in pASD more than in controls, *F* (1,49) = 12.54, *p* = .001. A marginally significant trend towards the same pattern was also seen in LOC (*F* (1.49) = 6.11, *p* = .017) and FFG (*F* (1,49) = 3.99, *p* = .051).

There was a marginally significant main effect of group on total early beta-band power in SMG (*F* (1,49) = 4.41, *p* = .041) and STG (*F* (1,49) = 4.38, *p* = .041). As with evoked early beta power, both groups demonstrated increased total power, but pASD showed greater increases than controls. A significant main effect of hemisphere was found in early total beta power in FFG, with greater total beta seen in the left hemisphere, *F* (1,49) = 12.36, *p* = .001.

A marginally significant effect of group was found in early beta-band phase-locking factor (PLF) in FFG (*F* (1,48) = 5.28, *p* = .026), with pASD showing greater PLF than controls. A significant main effect of hemisphere was found in early beta PLF in SMG, with greater phase-locking in the right hemisphere compared to left, *F* (1,48) = 7.57, *p* = .008.

#### Late beta-band activity

There were no significant group differences in late (200–800 ms) evoked or total beta-band power. However, there was a significant effect of hemisphere in LOC (*F* (1,49) = 15.71, *p* < .001) and auditory cortex (*F* (1,49) = 7.32, *p* = .009), with greater decreases in late total power seen in left compared to right hemisphere.

### Regression analyses

Multivariate regression analyses were used to investigate relationships between gamma-band activity in each ROI and the language measures taken prior to MEG recording, to assess if gamma-band abnormalities may be related to language aspects of the BAP. This was assessed for evoked and total gamma-band activity in the early tGBR (30–150 ms post-stimulus) and later gamma activity (200–800 ms post-stimulus). This was investigated for evoked and total beta-band activity in the same time windows. Results are presented separately for each of the language measures in which relationships were found.

#### Nonword repetition

**Figure 6:**
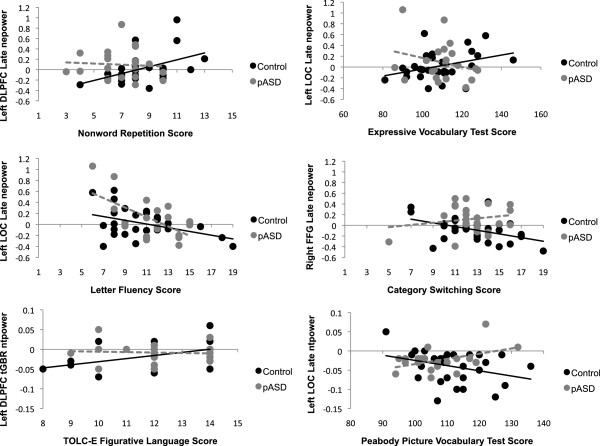
**Gamma regression results.** Representative plots of the regression results assessing the relationships between gamma-band activity and language measure scores. nepower: normalized evoked power; ntpower: normalized total power; tGBR: transient gamma band response (30–150 ms post-stimulus), late: late gamma band response (200–800 ms post-stimulus); DLPFC: dorsolateral prefrontal cortex; LOC: lateral occipital cortex; FFG: fusiform gyrus; TOLC-E: Test of Language Competence-Expanded Edition.

There was a marginally significant relationship between Nonword Repetition score (subtest of the CTOPP) and early tGBR evoked activity in right DLPFC in pASD, such that as score increased, evoked activity decreased, B = −2.07, *p* = .044. In left DLPFC, there was a marginally significant group interaction (*p* = .056) for later evoked gamma activity, with the control group showing a positive relationship between Nonword Repetition score and evoked gamma, B = 3.22, *p* = .013 (i.e., higher scores were related to a greater increase in evoked activity). There was no such relationship in the pASD group (B = −.47, *p* = .741) (see Figure 6).

In left auditory cortex, there was a marginally significant interaction between Nonword Repetition score and tGBR evoked gamma (*p =* .049), with a trend toward higher scores being associated with reduced evoked gamma in pASD (B = −1.80, *p* = .082), and no such relationship for the control group (B = 1.46, *p* = .250). Additionally, a marginally significant relationship in left SMG in controls suggested higher scores were associated with increased tGBR evoked activity, B = 2.36, *p* = .043. There was also a marginally significant interaction in total gamma activity in left LOC, *p* = .039. Controls exhibited a trend towards a negative relationship between Nonword Repetition score and total gamma (B = −18.36, *p* = .085), while pASD demonstrated a positive but non-significant slope (B = 16.67, *p* = .197).

**Figure 7:**
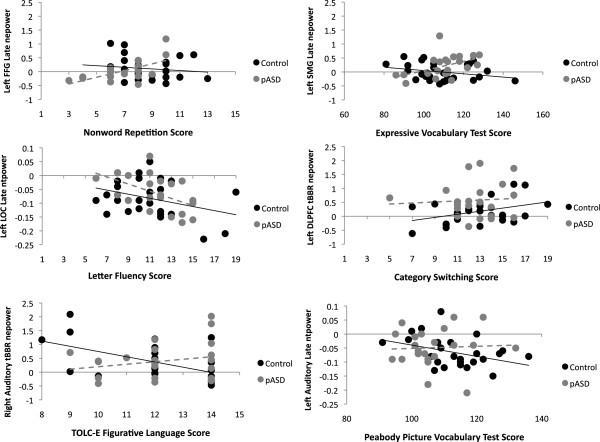
**Beta regression results.** Representative plots of the regression results assessing the relationships between beta-band activity and language measure scores. nepower: normalized evoked power; ntpower: normalized total power; tBBR: transient beta band response (30–150 ms post-stimulus), late: late beta band response (200–800 ms post-stimulus); DLPFC: dorsolateral prefrontal cortex; LOC: lateral occipital cortex; FFG: fusiform gyrus; TOLC-E: Test of Language Competence-Expanded Edition.

In early total beta activity, there was a significant relationship between Nonword Repetition score and beta power for controls in left auditory cortex (B = −22.92, *p* = .007) and left FFG (B = −26.98, *p* = .017), such that higher scores were associated with reduced total beta activity. While the same pattern was seen for pASD in left auditory cortex (B = −3.86, *p* = .682) and left FFG (B = −13.60, *p* = .198), the relationship was not statistically significant. There were no significant associations with Nonword Repetition score in the late beta response (200–800 ms) for total activity, but there was a significant interaction in late evoked beta in left FFG, *p* = .007 (see Figure 7). This was such that higher Nonword Repetition scores were significantly associated with higher evoked activity for pASD (B = 3.18, *p* = .005), but not for controls (B = −.70, *p* = .417).

#### Figurative language

In left DLPFC, a significant relationship between early tGBR total activity and Figurative Language score (subtest of the TOLC-E) was found in the control group, such that higher scores were associated with increased total activity, B = 26.78, *p* = .010. While not significant, the opposite pattern was seen in pASD (B = −4.46, *p* = .757), with a trend towards an interaction between the two groups, *p* = .081 (see Figure 6). Additionally, a marginally significant group interaction was seen in right auditory cortex, *p* = .036. This reflected that while the slopes in each individual group were not significant, they were in opposite directions: pASD demonstrated a negative relationship between test score and total gamma (B = −21.34, *p* = .103), while controls showed a positive relationship (B = 15.40, *p* = .176).

A significant group interaction was seen in the early evoked beta response in right auditory cortex, *p* = .004 (see Figure 7). Controls showed a significant association between higher Figurative Language scores and reduced evoked beta activity (B = −1.62, *p* = .002), while pASD did not show this relationship (B = .256, *p* = .315).

#### Letter fluency

In left LOC, there were significant relationships between both evoked and total early tGBR and Letter Fluency score (part of the Verbal Fluency subtest of the DK-EFS) for the control group. For both evoked (B = −4.77, *p* = .002) and total (B = −40.70, *p* = .012) gamma, higher scores were associated with reduced gamma activity in controls. This was not observed in the pASD group for total gamma (B = .95, *p* = .960), but there was a trend in evoked gamma (B = −2.40, *p* = .065). Similarly, in the later gamma-band response, higher scores were associated with reduced evoked gamma in left LOC in the control group, B = −5.00, *p* = .012. In this time window, the same relationship was also seen in pASD, B = −4.18, *p* = .011. Additionally, pASD (but not controls, B = −.32, *p* = .885) showed the same relationship between Letter Fluency score and late evoked gamma in right LOC, B = −5.26, *p* = .006. A similar association was seen in right FFG, with increased scores associated with reduced late evoked gamma in the pASD group (B = −5.89, *p* = .015), but not significantly in the control group (B = −2.87, *p* = .231). In left FFG, a comparable association was found for late total gamma, such that higher scores in both the control (B = −27.90, *p* = .039) and pASD (B = −45.80, *p* = .050) groups were associated with decreased total gamma (see Figure 6).

In late total beta power, both controls and pASD showed a similar pattern of higher Letter Fluency scores being associated with reduced total beta power (see Figure 7). This relationship was significant for controls in left FFG (B = −18.5, *p* = 004) and marginally in right DLPFC (B = −20.35, *p* = .012), right FFG (B = −17.43, *p* = .030), left LOC (B = −17.05, *p* = .035), and right auditory cortex (B = −18.67, *p* = .047). The same relationship was marginally significant for pASD in right FFG (B = −23.96, *p* = .034) and left LOC (B = −20.11, *p* = .036), but not for right auditory cortex (B = −22.91, *p* = .052), left FFG (B = −17.39, *p* = .069), or right DLPFC (B = −10.95, *p* = .182).

#### Category switching

A negative relationship was seen in the control group in both right (B = −.606, *p* = .009) and left (B = −4.33, *p* = .034) FFG, with greater Category Switching (part of the Verbal Fluency subtest of the DK-EFS) accuracy associated with reduced late evoked gamma activity. This relationship was not seen in pASD in either left (B = −1.34, *p* = .287) or right FFG (B = 1.84, *p* = .407), but there was a significant group interaction in right FFG (*p* = .015) (see Figure 6).

In the early evoked beta response, a significant group interaction was seen in left auditory cortex (*p* = .004). There was a marginally significant relationship between greater Category Switching accuracy and reduced evoked beta activity for pASD (B = −2.32, *p* = .015), but not for controls (B = 2.26, *p* = .072). Similarly, there was a marginally significant group interaction in left DLPFC (*p* = .020), with controls showing a marginally significant association between greater accuracy and higher evoked beta activity (B = 2.85, *p* = .022) not seen in pASD (B = −.80, *p* = .386) (see Figure 7).

#### Expressive vocabulary test

In left LOC, there was a marginally significant group interaction (*p* = .026), such that while higher expressive vocabulary scores were associated with increased late evoked gamma in the control group (B = 19.64, *p* = .041), there was no significant association in the pASD group (B = −8.28, *p* = .291) (see Figure 6). Conversely, in right LOC, higher scores in the control group were associated with *decreased* late evoked gamma activity, B = −22.19, *p* = .036. Again, no such relationship was seen in the pASD group, B = −4.46, *p* = .603. The same was seen in left FFG, with higher scores associated with decreased late evoked activity in the control group (B = −22.03, *p* = .025), but not pASD (B = −9.19, *p* = .493). A similar relationship was seen in left DLPFC in late total gamma in the control group, with higher scores associated with decreased gamma, B = −222.31, *p* = .011. While there was a marginally significant group interaction (*p* = .029), there was no significant relationship between expressive vocabulary scores and late total gamma in left DLPFC for the pASD group, B = 49.77, *p* = .572.

In the late evoked beta response, there was a group interaction in left SMG, *p* = .014 (see Figure 7). Higher expressive vocabulary scores were associated with reduced evoked beta in controls (B = −14.17, *p* = .091) while higher scores were associated with *increased* evoked beta in pASD (B = 13.41, *p* = .062).

#### Peabody picture vocabulary test (PPVT-III)

Higher PPVT score was marginally associated with reduced early total tGBR in left auditory cortex in the control group, B = −123.94, *p* = .045. This was not seen in pASD (B = −93.13, *p* = .263). A significant group interaction was found for late total gamma activity in left DLFPC (*p* = .015), left LOC (*p* = .010; see Figure 6) and left SMG (*p* = .046). In general, while not all slopes were statistically significant, the control group showed a negative relationship between PPVT score and late total gamma (DLPFC: B = −125.41, *p* = .052; LOC: B = −79.03, *p* = .071; SMG: B = −70.58, *p* = .157), while the pASD group showed a positive relationship (DLPFC: B = 104.97, *p* = .117; LOC: B = 141.13, *p* = .048; SMG: B = 107.36, *p* = .141).

Higher PPVT scores were marginally associated with reduced early total beta activity in right DLPFC for both controls (B = −105.97, *p* = .023) and pASD (B = −107.44, *p* = .034). Increased late evoked beta activity in left auditory cortex was marginally associated with lower PPVT scores for the control group (B = 13.00, *p* = .036) but not for pASD (B = .969, *p* = .860) (see Figure 7).

### Lateralization index

For consistency with previous studies investigating lateralization of the evoked response during a continuous word recognition task [39], lateralization of evoked activity was assessed for both gamma and beta activity in each ROI during the early (0–200 ms) and late (200–800 ms) post-stimulus time windows. A marginally significant group difference in lateralization of late evoked gamma activity was seen in LOC, (*t* (48) = 2.19, *p* = .033), with pASD demonstrating greater left lateralization compared to controls. Across both groups, significant left gamma lateralization 200–800 ms post-stimulus was seen in LOC and DLPFC, *p* < .001, with significant right lateralization in auditory cortex, *p* = .001. Bilateral activation was seen in SMG and FFG. There were no group differences in evoked gamma lateralization during the 0–200 ms post-stimulus window. As seen in previous studies [39,47], gamma activation during this earlier time window was largely bilateral, with bilateral activation in auditory cortex, LOC, SMG, and FFG across groups. However, activity in DLPFC was left lateralized during this time, *p* < .001.

In addition, lateralization of total gamma power was explored, as this has not previously been investigated in pASD. During the 200–800 ms post-stimulus time window, there were no significant group differences in lateralization of total gamma. Across groups, total gamma activity was left lateralized in both SMG and DLPFC (*p* < .001), with bilateral activation in auditory cortex, FFG, and LOC. In the 0–200 ms post-stimulus time window, a marginally significant group difference was found in SMG, such that pASD had greater left lateralization than controls, *t* (49) = 2.18, *p* = .034. Across groups, total gamma power was left lateralized in DLPFC (*p* < .001) and SMG (*p* < .001), right lateralized in auditory cortex (*p* < .001), and bilateral in FFG, and LOC.

In the beta-band, a marginally significant group difference in lateralization of late evoked activity was found in DLPFC (*t* (49) = 2.39, *p* = .021). In DLPFC, both controls and pASD showed left lateralization of activity, but pASD showed greater lateralization than controls. Similarly, in auditory cortex, both groups had right lateralized beta activity, but the pASD group was more lateralized than controls t (49) = 2.06, *p* = .044. Across groups, late evoked beta was right lateralized in FFG and auditory cortex (*p* < .001), left lateralized in DLPFC and LOC (*p* < .001), and bilateral in SMG. As with gamma activity, there were no group differences in evoked beta activity in the 0–200 ms post-stimulus time window. Across groups, early evoked beta activity was left lateralized in FFG (*p* = .002), DLPFC (*p* < .001), and SMG (*p* = .003), right lateralized in auditory cortex (*p* < .001), and bilateral in LOC.

There was a significant group difference between lateralization of late *total* beta activity in SMG (t (49) = 2.63, *p* = .011), with controls showing bilateral activation and pASD demonstrating left lateralized activity. Across groups, late total beta activity was left lateralized in DLPFC and LOC (*p* < .001), right lateralized in auditory cortex and FFG (*p* < .001), and bilateral in SMG. In the early time window, there was a marginally significant group difference in lateralization of total beta activity, *t* (49) = 2.18, *p* = .034. While both groups showed left lateralization, the pASD group was more lateralized than controls. Across groups, early total beta activity was left lateralized in FFG, DLPFC, and SMG (*p* < .001), but right lateralized in auditory cortex and LOC (*p* < .001).

## Discussion

As has been seen in several prior studies of language and gamma [13,36-38], the language task in the current study elicited strong gamma event-related desynchronization (ERD; see Figures 1 and 2). ERD of gamma activity was seen in both controls and pASD, but greater ERD was seen in the control group. While previous studies with simple tone/click stimuli found evoked gamma-band power to be reduced in those with ASD [26,27] and in pASD [27,30] compared to control groups, the current study found pASD to show *increased* evoked power overall compared to controls. Gamma-band abnormalities in response to language stimuli have not previously been investigated in first-degree relatives of individuals with ASD, but this finding is consistent with previous research finding adults with ASD to show greater increases in evoked gamma activity in response to a sentence context evaluation task compared to controls [29].

A likely explanation for the disparity between the current results and previous results showing reduced evoked gamma in pASD compared to controls is that the complexity of the stimuli and the task demands differed: while previous studies of gamma-band abnormalities in pASD used pure tone and amplitude-modulated tone stimuli [27,30], the current study used spoken language stimuli in the context of a word recognition task. Rather than listening passively to a simple auditory stimulus, higher order cognitive processes including language and sustained attention were necessary for word recognition. While this has not been studied in pASD previously, these findings are consistent with those of Braeutigam et al. [29], who reported increased evoked gamma in both control adults and adults with ASD during a task involving evaluation of sentence context, but found the ASD group to show greater increases in evoked gamma that were more widespread across the time window than controls.

Both the early transient gamma-band response (tGBR; 30–150 ms post-stimulus) and the later gamma-band response (200–800 ms post-stimulus) were assessed, with similar results found in both time windows. During the earlier tGBR, there were significant group differences in supramarginal gyrus (SMG) and lateral occipital cortex (LOC), and marginally significant group differences in auditory cortex and fusiform gyrus (FFG), in which pASD showed increased evoked gamma activity and controls showed decreased evoked gamma. The same pattern was seen in the later evoked gamma-band responses in both SMG and LOC. While both groups showed increased evoked power in the later gamma-band response in auditory cortex, the pASD group still showed a greater increase than controls. Additionally, in FFG and LOC in both the early and late gamma response, both groups showed decreases in total power. However, controls exhibited greater reduction than the pASD group. In both the early and late time windows, there were no hemispheric differences in evoked power in controls, but there were differences between evoked power in left and right hemispheres in pASD; pASD showed increased evoked power in the right hemisphere in FFG, but evoked power was reduced in the left hemisphere. There were no significant group differences in phase-locking factor (PLF), but in both auditory cortex and SMG, PLF was greater in the left hemisphere than the right. This is of note as the opposite has been seen in non-language auditory tasks [30] and suggests that PLF in this study may be specifically related to language processing.

The cognitive processes involved in the word recognition task could explain the reduction in total gamma power seen in both groups, and the increase in evoked gamma seen in pASD relative to controls. While increases in evoked gamma activity are often seen when attention is increased [5,72-75], some studies have found reduced gamma activity following repetition or priming during cognitive tasks [76,77]. Given that the current results are in response to stimuli repeated multiple times throughout the task, it may be that total gamma activity was reduced due to word repetition. Some hypothesize that reduced gamma activity following repeated presentations of stimuli reflects the need for less neural representation as a result of a more “sharpened” neural network [76,77]. This could explain why pASD showed increases in evoked gamma activity, while controls showed decreased evoked gamma, and why pASD also showed less reduction in total gamma power compared to controls. The reduction in total and evoked gamma activity in controls could be a result of their better formation of target word representations. Conversely, the increased evoked gamma and attenuated reduction in total gamma seen in pASD may reflect that they were less able to form a neural representation of target words.

The current study also investigated group differences in neural activity in the beta-band (13–30 Hz), as beta activity may also be involved in language processing [12,37,44,45] and increased beta activity has previously been observed in children with ASD compared to healthy controls [46]. Indeed, while both groups demonstrated increased early evoked beta activity, the current study found a significantly greater increase in pASD compared to controls in SMG, with a similar trend in both LOC and FFG. While there were no group differences in early total beta activity, there was a significant main effect of hemisphere in FFG, with greater increases in total activity seen in left compared to right hemisphere, across groups. Similarly to gamma activity, an overall decrease in total beta activity was observed, but there were no significant group differences in total beta. Interestingly, both groups demonstrated early increases in beta activity, followed by a later decrease. This could indicate cognitive differences between earlier and later stages of language processing; previous studies have suggested increased beta activity to be associated with top-down cognitive processing and decreased beta to be associated with bottom-up cognitive processing [42]. The group differences seen in beta activity also suggest disparate cognitive function during the language task, as with gamma differences. However, given the dearth of research on beta activity in ASD, further investigation is warranted.

As language deficits are sometimes seen as part of the BAP [35,40,41] and the current sample of pASD did indeed show worse performance than controls on measures of phonology and receptive language, it is reasonable that this task would involve greater cognitive effort for pASD compared to controls. This hypothesis is also consistent with the pattern of group differences in neural activity being similar across regions of interest in the brain. If gamma and/or beta activity reflect task-related activation of language networks, these brain regions would be expected to be synchronously active or inhibited [78]. Additionally, the use of word stimuli in the continuous word recognition task likely explains why a later gamma-band response was seen in addition to the early tGBR seen in previous studies using tone/click-train stimuli. In their study comparing gamma-band activation between words and pseudowords using MEG, Pulvermuller et al. [12] saw activation even after 700 ms post-stimulus and suggested the use of word stimuli likely accounted for the prolonged effect seen compared to tone or click stimuli.

An alternative hypothesis is that the later reduction in gamma-band activity could be due to sustained attention. Brookes et al. [79] found reductions in evoked gamma activity during visual working memory tasks and found this decrease to be larger during more demanding phases of the tasks. They suggested that this reduction reflects gamma activity being suppressed during working memory maintenance to prevent attention shifting to non task-related distractions. Given that attention dysfunction is common in ASD [18,23] and may be part of the BAP [80], the lack of evoked gamma reduction and attenuation of total gamma reduction in pASD relative to the control group could reflect attentional dysfunction.

There were a number of interesting relationships between gamma activity and scores on language measures in the current study. For figurative language, higher scores were related to reduced evoked and increased total gamma power in the control group. This relationship was not significant for the pASD group. Receptive language scores showed a negative relationship with late total gamma in the control group, with higher scores associated with reduced evoked gamma. This fits well in the context of the hypothesis that reduced gamma may reflect a sharpened neural representation of the target words. Given that receptive language skills are important in a spoken word recognition task, this suggests that those with better receptive language performance may have shown a greater reduction in total gamma as a result of being better equipped to form a neural representation of the words. However, as with figurative language, this relationship was not significant in pASD. Similarly, higher scores on expressive language, letter fluency, and category switching accuracy in the control group were largely associated with decreased total and evoked gamma activity, with few significant relationships observed in the pASD group. That the majority of the significant relationships between language performance and gamma activity were seen in the control group, but not in pASD may suggest that language performance and gamma activity are more clearly linked in controls than in pASD overall. It should be noted, however, that these analyses were explorative in nature and should be interpreted with caution. Future studies should further explore the relationship between language performance and gamma activity by directly measuring gamma during these tasks.

There were also some relationships of note between beta-band activity and the language measures. While both pASD and controls showed an association between higher nonword repetition scores and total beta activity, this relationship was stronger for the control group. Similarly, higher figurative language scores were associated with increased total beta activity in the control group, but not in the pASD group. Higher expressive vocabularly scores in the control group were associated with reduced evoked activity in controls, but increased evoked beta in pASD. In sum, similarly to gamma-band activity, the relationship between beta-band activity and language performance appears to be different between controls and pASD and merits further investigation.

As the continuous word recognition task has been used previously to assess lateralization of language function [39], we also investigated lateralization of activity. Consistent with previous findings, we found early evoked gamma activity (0–200 ms post-stimulus) to be largely bilateral (with the exception of left lateralization in DLPFC), with later evoked activity (200–800 ms post-stimulus) showing greater left lateralization. Results were similar for total gamma activity, although greater lateralization of function was seen earlier. Evoked and total beta activity were more lateralized than gamma activity, but this differed among brain areas; DLPFC activity was consistently left lateralized, auditory cortex was consistently right lateralized, FFG and SMG were left lateralized in the early time window and right lateralized in the late time window, and LOC was left lateralized in the late time window and right lateralized in the early time window. This difference in lateralization supports that gamma and beta activity may play different roles in language processing [43,45]. Interestingly, in areas for which there were group differences (LOC and DLPFC for evoked gamma; auditory cortex and DLPFC for evoked beta; SMG and DLPFC for total gamma; SMG for total beta), pASD showed greater left lateralization compared to controls. Previous studies have suggested that individuals with ASD show atypical language lateralization, with lateralization reduced [81,82] or more rightward [47] compared to controls. Since greater left lateralization was seen in pASD, it may suggest a compensatory mechanism not seen in individuals with ASD.

A potential limitation of the current study is that is not possible to parse out attention effects from language effects. As such, it is difficult to tell if these results differ from those in studies using simple tone stimuli as a result of the language stimulus itself and the cognitive processes required to distinguish repeated from novel words, or as a result of participants actively attending to stimuli during the task. Future studies will adjust for these variables separately to assess the impact of each on neural activity by varying the loading on language and attention processes independently. Furthermore, standardized measures of attention can be taken in the future to address the potential relationship between neural activity and attention dysfunction. It has been suggested that attention relies on gamma synchronization to form a working memory representation of an object [77], so it will be important to assess the relationship between them [29].

## Conclusions

In conclusion, this study extends the findings of previous research showing gamma-band abnormalities in pASD in response to simple tone stimuli by also revealing abnormalities in response to language stimuli. The results of the current study are consistent with at least one other prior study using a language task in persons with ASD [29] and are supportive of language-related neural activity as a heritable biomarker of ASD. The current study also found group differences in beta-band activity, also thought to be involved in language processing, and suggests the possible use of beta activity as an ASD biomarker. Future studies will further assess whether these potential neural biomarkers are better characterized in response to simple auditory stimuli or to more complex stimuli involving higher order cognitive processes, such as attention and language processing. The current results also suggest a possible relationship between language abilities and neural activity, which should be further explored in individuals with ASD who have more prominent language difficulties than the pASD group in the current study. The determination of distinct patterns of oscillatory activity for those with and without language dysfunction could be helpful for assessment of efficacy of language-focused ASD interventions and could also prove useful in ASD subtyping in genetic research studies.

## Competing interests

The authors declare that they have no competing interests.

## Authors’ contributions

KLM conducted the data analyses and wrote the first draft of the manuscript. SH contributed to participant recruitment, measure selection and diagnosis of probands. EW and GLS were involved in data acquisition and analyses. DCR conceived of the study, participated in study design and coordination, contributed to the data analytic strategy, and helped to draft the manuscript. All authors read and approved the final manuscript.

## Pre-publication history

The pre-publication history for this paper can be accessed here:

http://www.biomedcentral.com/1471-244X/12/213/prepub

## Supplementary Material

Additional file 1 **Figure S1.** Statistical results of an independent-samples t-test comparison between sensor-level neural activity in parents of a child with an autism spectrum disorder (pASD) and healthy adult control participants. Time and frequency windows of interested are represented as follows: A) early (30-150 ms) evoked gamma-band activity (30-50 Hz), B) late (200-800 ms) evoked gamma-band activity, C) early evoked beta-band activity (13-30 Hz), and D) late evoked beta-band activity. Sensors demonstrating a statistically significant group difference (uncorrected *p* < .01) are indicated with an asterisk overlayed on the sensor. Statistical values above 0 indicate areas in which pASD showed greater evoked activity compared to controls; those below 0 indicate areas in which pASD showed less activity than controls (pASD > HC shown in warmer colors; HC > pASD shown in cooler colors). Click here for file
