# Supplementary material for: Abnormalities in gamma-band responses to language stimuli in first-degree relatives of children with autism spectrum disorder: an MEG study
Source: BMC Psychiatry. 2012 Nov 29;12:213. doi: 10.1186/1471-244X-12-213 (PMC3557147; doi:10.1186/1471-244X-12-213)
Supplement: Additional file 1 — Figure S1. Statistical results of an independent-samples t-test comparison between sensor-level neural activity in parents of a child with an autism spectrum disorder (pASD) and healthy adult control participants. Time and frequency windows of interested are represented as follows: A) early (30-150 ms) evoked gamma-band activity (30-50 Hz), B) late (200-800 ms) evoked gamma-band activity, C) early evoked beta-band activity (13-30 Hz), and D) late evoked beta-band activity. Sensors demonstrating a statistically significant group difference (uncorrected p < .01) are indicated with an asterisk overlayed on the sensor. Statistical values above 0 indicate areas in which pASD showed greater evoked activity compared to controls; those below 0 indicate areas in which pASD showed less activity than controls (pASD > HC shown in warmer colors; HC > pASD shown in cooler colors). [file 1471-244X-12-213-S1.docx]

**Supplemental Data and Figure**

To address reviewer concern that a sensor space analysis would be more appropriate than the source space analysis used in the current paper, we also performed the analyses in sensor space. As with the source space analyses, sensor space analyses utilized epoched datasets with artifacts removed, and only including the target word trials. Time-frequency analyses were conducted using FieldTrip (Oostenveld et al., 2011), a software toolbox for MEG and EEG data analysis implemented in MATLAB (2009b; MathWorks, Inc., Matick, MA). Data were transformed to the time-frequency domain using a Morlet wavelet (wave number 6) decomposition. Statistical analyses were performed using Fieldtrip routines. An independent-samples t-test was conducted, using a false discovery rate (FDR) of q = .05 for multiple comparison correction. Analyses were conducted for both the gamma-band frequency range (30-50 Hz) and the beta-band frequency range (13-30 Hz), for both the early (30-150 ms) and late (200-800 ms) time windows used in the source space analyses.


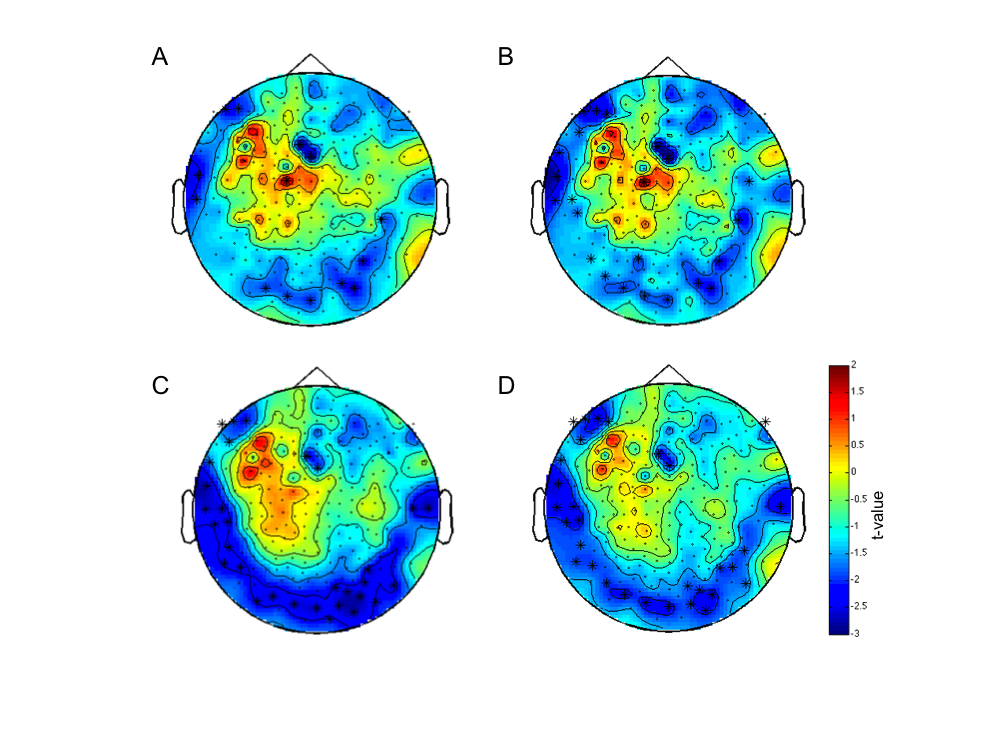
 A similar pattern of was seen in the sensor-level analyses as was seen in source space, although none of the sensor-level results survived multiple comparison correction. Supplemental Figure 1 shows the results of the sensor-level analyses for evoked gamma and beta activity. Sensors in which there was a significant group difference (p < .01) prior to multiple comparison correction have been indicated with an asterisk over the sensor.

**Supplemental Figure 1 caption:** Statistical results of an independent-samples t-test comparison between sensor-level neural activity in parents of a child with an autism spectrum disorder (pASD) and healthy adult control participants. Time and frequency windows of interested are represented as follows: A) early (30-150 ms) evoked gamma-band activity (30-50 Hz), B) late (200-800 ms) evoked gamma-band activity, C) early evoked beta-band activity (13-30 Hz), and D) late evoked beta-band activity. Sensors demonstrating a statistically significant group difference (uncorrected p < .01) are indicated with an asterisk overlayed on the sensor. Statistical values above 0 indicate areas in which pASD showed greater evoked activity compared to controls; those below 0 indicate areas in which pASD showed less activity than controls (pASD > HC shown in warmer colors; HC > pASD shown in cooler colors).

In source space analyses, pASD showed greater evoked gamma-band activity in left hemipsphere compared to controls, overall. This pattern is also reflected in the sensor space analyses seen in Supplemental Figure 1. The advantage of source space analyses compared to sensor space analyses is that the signal to noise ratio (SNR) has been shown to be higher when performing source space analyses (Ross et al., 2000). As such, it is not surprising that the sensor space analyses, while showing similar results, did not demonstrate statistical significance following multiple comparison correction.
